# Supplementary material for: Calculation and realization of new method grey residual error correction model
Source: PLoS One. 2021 Jul 19;16(7):e0254154. doi: 10.1371/journal.pone.0254154 (PMC8289091; doi:10.1371/journal.pone.0254154)
Supplement: S1 File — (ZIP) [file pone.0254154.s001.zip › Experimental data and code/ExperimentCode.docx]

clear

%%%%%%%%%%%%%%%%%%%%%%%%%%%Experiment with petroleum data%%%%%%%%%%%%%%%%%%%%%%%%%%%%%%%%%%

%%%%%%%%%%%%%%%%%%%%%%%%%%%%%%%Mean model%%%%%%%%%%%%%%%%%%%%%%%%%%%%%%%%%%%%%

syms aCS bCS;

ParatMatrixCS = [aCS , bCS]';

OriValueCS = [17.91 , 17.89 , 17.39 , 17.65 , 17.61 , 17.35 , 16.75 , 17.86 , 17.46 , 17.66];

AccValueCS = cumsum(OriValueCS);

n = length(OriValueCS);

for k = 2 : (n-1)

ZCS(k) = (AccValueCS(k - 1) + AccValueCS(k)) * 0.5;

end

YnCS = OriValueCS;

YnCS(1) = [];

YnCS = YnCS';

ECS = [-ZCS;ones(1,n-1)]';

ParatMatrixCS = (ECS'*ECS)\(ECS'*YnCS);

ParatMatrixCS = ParatMatrixCS';

aCS = ParatMatrixCS(1);

bCS = ParatMatrixCS(2);

FCS = [];

FCS(1) = OriValueCS(1);

for k = 2 : (n)

FCS(k) =(OriValueCS(1) - bCS/aCS)/exp(aCS*(k - 1)) + bCS/aCS;

end

PreValueMEAN = [];

PreValueMEAN(1) = OriValueCS(1);

for k = 2:(n)

PreValueMEAN(k) = FCS(k) - FCS(k - 1);

end

for k = 2:(n)

ResidualError(k) = OriValueCS(k) - PreValueMEAN(k);

end

ResidualErrorABSMEAN = abs(ResidualError);

RelativeErrorMEAN = [];

RelativeErrorMEAN(1) = 0;

for k = 2:(n)

RelativeErrorMEAN(k) = ResidualErrorABSMEAN(k) / OriValueCS(k) ;

end

E0 = ResidualError';

%%%%%%%%%%%%%%%%%%%%%%%%%Mexican straw hat wavelet analysis mode%%%%%%%%%%%%%%%%%%%%%%%%%%%%%%

WLI = cwt(ResidualError,1:10,'mexh');

PXP = WLI;

CXB = (inv(PXP'*PXP))*PXP'*E0;

for x = 1:(n)

for i = 1:(n)

JieGuoHang(x,i) = CXB(i) * WLI(x,i);

end

ResidualErrorXBCL(x) = sum(JieGuoHang(x,:));

end

PreValueXBFX = PreValueMEAN + ResidualErrorXBCL;

ResidualErrorXBFX = OriValueCS - PreValueXBFX;

ResidualErrorABSXBFX = abs(ResidualErrorXBFX);

RelativeErrorXBFX = [];

RelativeErrorXBFX(1) = 0;

for k = 2:(n)

RelativeErrorXBFX(k) = ResidualErrorABSXBFX(k) / OriValueCS(k) ;

end

%%%%%%%%%%%%%%%%%%%%%%%%%%%%%Fourier transform model%%%%%%%%%%%%%%%%%%%%%%%%%%%%%%%%%

PFLY = [1/2 , cos(1*1*2*pi/(n-1)) , sin(1*1*2*pi/(n-1)) , cos(2*1*2*pi/(n-1)) , sin(2*1*2*pi/(n-1)) , cos(3*1*2*pi/(n-1)) , sin(3*1*2*pi/(n-1)) ;

1/2 , cos(1*2*2*pi/(n-1)) , sin(1*2*2*pi/(n-1)) , cos(2*2*2*pi/(n-1)) , sin(2*2*2*pi/(n-1)) , cos(3*2*2*pi/(n-1)) , sin(3*2*2*pi/(n-1)) ;

1/2 , cos(1*3*2*pi/(n-1)) , sin(1*3*2*pi/(n-1)) , cos(2*3*2*pi/(n-1)) , sin(2*3*2*pi/(n-1)) , cos(3*3*2*pi/(n-1)) , sin(3*3*2*pi/(n-1)) ;

1/2 , cos(1*4*2*pi/(n-1)) , sin(1*4*2*pi/(n-1)) , cos(2*4*2*pi/(n-1)) , sin(2*4*2*pi/(n-1)) , cos(3*4*2*pi/(n-1)) , sin(3*4*2*pi/(n-1)) ;

1/2 , cos(1*5*2*pi/(n-1)) , sin(1*5*2*pi/(n-1)) , cos(2*5*2*pi/(n-1)) , sin(2*5*2*pi/(n-1)) , cos(3*5*2*pi/(n-1)) , sin(3*5*2*pi/(n-1)) ;

1/2 , cos(1*6*2*pi/(n-1)) , sin(1*6*2*pi/(n-1)) , cos(2*6*2*pi/(n-1)) , sin(2*6*2*pi/(n-1)) , cos(3*6*2*pi/(n-1)) , sin(3*6*2*pi/(n-1)) ;

1/2 , cos(1*7*2*pi/(n-1)) , sin(1*7*2*pi/(n-1)) , cos(2*7*2*pi/(n-1)) , sin(2*7*2*pi/(n-1)) , cos(3*7*2*pi/(n-1)) , sin(3*7*2*pi/(n-1)) ;

1/2 , cos(1*8*2*pi/(n-1)) , sin(1*8*2*pi/(n-1)) , cos(2*8*2*pi/(n-1)) , sin(2*8*2*pi/(n-1)) , cos(3*8*2*pi/(n-1)) , sin(3*8*2*pi/(n-1)) ;

1/2 , cos(1*9*2*pi/(n-1)) , sin(1*9*2*pi/(n-1)) , cos(2*9*2*pi/(n-1)) , sin(2*9*2*pi/(n-1)) , cos(3*9*2*pi/(n-1)) , sin(3*9*2*pi/(n-1)) ;

1/2 , cos(1*10*2*pi/(n-1)) , sin(1*10*2*pi/(n-1)) , cos(2*10*2*pi/(n-1)) , sin(2*10*2*pi/(n-1)) , cos(3*10*2*pi/(n-1)) , sin(3*10*2*pi/(n-1))];

CFLY = (inv(PFLY'*PFLY))*PFLY'*E0;

for k = 1:((n-1)/2)-1

Xsa(k) = CFLY(2*k);

Xsb(k) = CFLY(2*k+1);

end

ResidualErrorFYC = [];

ResidualErrorFYC(1) = 0;

for k = 2:(n)

for i = 1:((n-1)/2)-1

FuliyeQH(i) = Xsa(i) * cos(i*k*2*pi/(n-1)) + Xsb(i) * sin(i*k*2*pi/(n-1));

end

ResidualErrorFYC(k) = (1/2)*CFLY(1) + sum(FuliyeQH);

end

PreValueFLY = PreValueMEAN + ResidualErrorFYC;

ResidualErrorFLY = OriValueCS - PreValueFLY;

ResidualErrorABSFLY = abs(ResidualErrorFLY);

RelativeErrorFLY = [];

RelativeErrorFLY(1) = 0;

for k = 2:(n)

RelativeErrorFLY(k) = ResidualErrorABSFLY(k) / OriValueCS(k) ;

end

%%%%%%%%%%%%%%%%%%%%%%%%%%%%%%%%mapping%%%%%%%%%%%%%%%%%%%%%%%%%%%%%%%%%%%%%%%%

X = 0.5:1:9.5;

Date = {'May 09' ,'May 12', 'May 13' , 'May 14' , 'May 16' , 'May 19' , 'May 20' ,'May 21' , 'May 22' , 'May 23'};

OriValue = [17.91 , 17.89 , 17.39 , 17.65 , 17.61 , 17.35 , 16.75 , 17.86 , 17.46 , 17.66];

MeanPreValue = PreValueMEAN;

ResidualPreValue1 = PreValueFLY;

ResidualPreValue2 = PreValueXBFX;

plot(X,OriValue,'bx-');hold on;

plot(X,MeanPreValue,'rh--');hold on;

plot(X,ResidualPreValue1,'ko-');hold on;

plot(X,ResidualPreValue2,'k<-.');hold on;

xlabel({'Date'},'FontSize',15,'FontWeight','bold','color','k');

ylabel({'Oil Price'},'FontSize',15,'FontWeight','bold','color','k');

set(gca,'XTick',X);

set(gca,'XTickLabel',Date);

set(gca,'XTickLabelRotation',30);

legend('Original Value','GM(1,1)','FGM(1,1)','WGM(1,1)');

clear

%%%%%%%%%%%%%%%%%%%%%%%%%%%Taiwan tourism data experiment%%%%%%%%%%%%%%%%%%%%%%%%%%%%%%

%%%%%%%%%%%%%%%%%%%%%%%%%%%%%%%Mean model%%%%%%%%%%%%%%%%%%%%%%%%%%%%%%%%%%%%%

syms aCS bCS;

ParatMatrixCS = [aCS , bCS]';

OriValueCS = [193510 , 190390 , 173730 , 147280 , 210610 , 175030 , 171540 , 175540 , 148140 , 171200 , 177530 , 181170];

AccValueCS = cumsum(OriValueCS);

n = length(OriValueCS);

for k = 2 : (n-1)

ZCS(k) = (AccValueCS(k - 1) + AccValueCS(k)) * 0.5;

end

YnCS = OriValueCS;

YnCS(1) = [];

YnCS = YnCS';

ECS = [-ZCS;ones(1,n-1)]';

ParatMatrixCS = (ECS'*ECS)\(ECS'*YnCS);

ParatMatrixCS = ParatMatrixCS';

aCS = ParatMatrixCS(1);

bCS = ParatMatrixCS(2);

FCS = [];

FCS(1) = OriValueCS(1);

for k = 2 : (n)

FCS(k) =(OriValueCS(1) - bCS/aCS)/exp(aCS*(k - 1)) + bCS/aCS;

end

PreValueMEAN = [];

PreValueMEAN(1) = OriValueCS(1);

for k = 2:(n)

PreValueMEAN(k) = FCS(k) - FCS(k - 1);

end

for k = 2:(n)

ResidualError(k) = OriValueCS(k) - PreValueMEAN(k);

end

ResidualErrorABSMEAN = abs(ResidualError);

RelativeErrorMEAN = [];

RelativeErrorMEAN(1) = 0;

for k = 2:(n)

RelativeErrorMEAN(k) = ResidualErrorABSMEAN(k) / OriValueCS(k) ;

end

E0 = ResidualError';

%%%%%%%%%%%%%%%%%%%%%%%%%Mexican straw hat wavelet analysis model%%%%%%%%%%%%%%%%%%%%%%%%%%%%%%

WLI = cwt(ResidualError,1:12,'mexh');

PXP = WLI;

CXB = (inv(PXP'*PXP))*PXP'*E0;

for x = 1:(n)

for i = 1:(n)

JieGuoHang(x,i) = CXB(i) * WLI(x,i);

end

ResidualErrorXBCL(x) = sum(JieGuoHang(x,:));

end

PreValueXBFX = PreValueMEAN + ResidualErrorXBCL;

ResidualErrorXBFX = OriValueCS - PreValueXBFX;

ResidualErrorABSXBFX = abs(ResidualErrorXBFX);

RelativeErrorXBFX = [];

RelativeErrorXBFX(1) = 0;

for k = 2:(n)

RelativeErrorXBFX(k) = ResidualErrorABSXBFX(k) / OriValueCS(k) ;

end

%%%%%%%%%%%%%%%%%%%%%%%%%%%%%Fourier transform model%%%%%%%%%%%%%%%%%%%%%%%%%%%%%%%%%

PFLY = [1/2 , cos(1*1*2*pi/(n-1)) , sin(1*1*2*pi/(n-1)) , cos(2*1*2*pi/(n-1)) , sin(2*1*2*pi/(n-1)) , cos(3*1*2*pi/(n-1)) , sin(3*1*2*pi/(n-1)) , cos(4*1*2*pi/(n-1)) , sin(4*1*2*pi/(n-1));

1/2 , cos(1*2*2*pi/(n-1)) , sin(1*2*2*pi/(n-1)) , cos(2*2*2*pi/(n-1)) , sin(2*2*2*pi/(n-1)) , cos(3*2*2*pi/(n-1)) , sin(3*2*2*pi/(n-1)) , cos(4*2*2*pi/(n-1)) , sin(4*2*2*pi/(n-1));

1/2 , cos(1*3*2*pi/(n-1)) , sin(1*3*2*pi/(n-1)) , cos(2*3*2*pi/(n-1)) , sin(2*3*2*pi/(n-1)) , cos(3*3*2*pi/(n-1)) , sin(3*3*2*pi/(n-1)) , cos(4*3*2*pi/(n-1)) , sin(4*3*2*pi/(n-1));

1/2 , cos(1*4*2*pi/(n-1)) , sin(1*4*2*pi/(n-1)) , cos(2*4*2*pi/(n-1)) , sin(2*4*2*pi/(n-1)) , cos(3*4*2*pi/(n-1)) , sin(3*4*2*pi/(n-1)) , cos(4*4*2*pi/(n-1)) , sin(4*4*2*pi/(n-1));

1/2 , cos(1*5*2*pi/(n-1)) , sin(1*5*2*pi/(n-1)) , cos(2*5*2*pi/(n-1)) , sin(2*5*2*pi/(n-1)) , cos(3*5*2*pi/(n-1)) , sin(3*5*2*pi/(n-1)) , cos(4*5*2*pi/(n-1)) , sin(4*5*2*pi/(n-1));

1/2 , cos(1*6*2*pi/(n-1)) , sin(1*6*2*pi/(n-1)) , cos(2*6*2*pi/(n-1)) , sin(2*6*2*pi/(n-1)) , cos(3*6*2*pi/(n-1)) , sin(3*6*2*pi/(n-1)) , cos(4*6*2*pi/(n-1)) , sin(4*6*2*pi/(n-1));

1/2 , cos(1*7*2*pi/(n-1)) , sin(1*7*2*pi/(n-1)) , cos(2*7*2*pi/(n-1)) , sin(2*7*2*pi/(n-1)) , cos(3*7*2*pi/(n-1)) , sin(3*7*2*pi/(n-1)) , cos(4*7*2*pi/(n-1)) , sin(4*7*2*pi/(n-1));

1/2 , cos(1*8*2*pi/(n-1)) , sin(1*8*2*pi/(n-1)) , cos(2*8*2*pi/(n-1)) , sin(2*8*2*pi/(n-1)) , cos(3*8*2*pi/(n-1)) , sin(3*8*2*pi/(n-1)) , cos(4*8*2*pi/(n-1)) , sin(4*8*2*pi/(n-1));

1/2 , cos(1*9*2*pi/(n-1)) , sin(1*9*2*pi/(n-1)) , cos(2*9*2*pi/(n-1)) , sin(2*9*2*pi/(n-1)) , cos(3*9*2*pi/(n-1)) , sin(3*9*2*pi/(n-1)) , cos(4*9*2*pi/(n-1)) , sin(4*9*2*pi/(n-1));

1/2 , cos(1*10*2*pi/(n-1)) , sin(1*10*2*pi/(n-1)) , cos(2*10*2*pi/(n-1)) , sin(2*10*2*pi/(n-1)) , cos(3*10*2*pi/(n-1)) , sin(3*10*2*pi/(n-1)), cos(4*10*2*pi/(n-1)) , sin(4*10*2*pi/(n-1))

1/2 , cos(1*11*2*pi/(n-1)) , sin(1*11*2*pi/(n-1)) , cos(2*11*2*pi/(n-1)) , sin(2*11*2*pi/(n-1)) , cos(3*11*2*pi/(n-1)) , sin(3*11*2*pi/(n-1)), cos(4*11*2*pi/(n-1)) , sin(4*11*2*pi/(n-1))

1/2 , cos(1*12*2*pi/(n-1)) , sin(1*12*2*pi/(n-1)) , cos(2*12*2*pi/(n-1)) , sin(2*12*2*pi/(n-1)) , cos(3*12*2*pi/(n-1)) , sin(3*12*2*pi/(n-1)), cos(4*12*2*pi/(n-1)) , sin(4*12*2*pi/(n-1))];

CFLY = (inv(PFLY'*PFLY))*PFLY'*E0;

for k = 1:((n-1)/2)-1

Xsa(k) = CFLY(2*k);

Xsb(k) = CFLY(2*k+1);

end

ResidualErrorFYC = [];

ResidualErrorFYC(1) = 0;

for k = 2:(n)

for i = 1:((n-1)/2)-1

FuliyeQH(i) = Xsa(i) * cos(i*k*2*pi/(n-1)) + Xsb(i) * sin(i*k*2*pi/(n-1));

end

ResidualErrorFYC(k) = (1/2)*CFLY(1) + sum(FuliyeQH);

end

PreValueFLY = PreValueMEAN + ResidualErrorFYC;

ResidualErrorFLY = OriValueCS - PreValueFLY;

ResidualErrorABSFLY = abs(ResidualErrorFLY);

RelativeErrorFLY = [];

RelativeErrorFLY(1) = 0;

for k = 2:(n)

RelativeErrorFLY(k) = ResidualErrorABSFLY(k) / OriValueCS(k) ;

end

%%%%%%%%%%%%%%%%%%%%%%%%%%%%%%%%mapping%%%%%%%%%%%%%%%%%%%%%%%%%%%%%%%%%%%%%%%%

X = 0.5:1:11.5;

Date = {'2006 Nov','2006 Dec','2007 Jan','2007 Feb','2007 Mar','2007 Apr','2007 May','2007 Jun','2007 Jul','2007 Aug','2007 Sep','2007 Oct'};

OriValue = [193510 , 190390 , 173730 , 147280 , 210610 , 175030 , 171540 , 175540 , 148140 , 171200 , 177530 , 181170];

MeanPreValue = PreValueMEAN;

ResidualPreValue1 = PreValueFLY;

ResidualPreValue2 = PreValueXBFX;

plot(X,OriValue,'bx-');hold on;

plot(X,MeanPreValue,'rh--');hold on;

plot(X,ResidualPreValue1,'ko-');hold on;

plot(X,ResidualPreValue2,'k<-.');hold on;

xlabel({'Date'},'FontSize',15,'FontWeight','bold','color','k');

ylabel({'Number of Tourists in Taiwan'},'FontSize',15,'FontWeight','bold','color','k');

set(gca,'XTick',X);

set(gca,'XTickLabel',Date);

set(gca,'XTickLabelRotation',30);

legend('Original Value','GM(1,1)','FGM(1,1)','WGM(1,1)');

clear

% %%%%%%%%%%%%%%%%%%%%%%%%%%%%%%The formula of posterior error%%%%%%%%%%%%%%%%%%%%%%%%%%%%%%%%

% OriValue = [17.91 , 17.89 , 17.39 , 17.65 , 17.61 , 17.35 , 16.75 , 17.86 , 17.46 , 17.66];

%

% ResidualError = [0,0.313846195051884,-0.164779357020379,0.116569097330647,0.0978915897224937,-0.140811848272641,-0.719541185124399,0.411703610663025,0.0329225705508733,0.254115725956726];

%

% RelativeError = [0,0.0175431076049125,0.00947552369294875,0.00660448143516413,0.00555886369804053,0.00811595667277469,0.0429576826939940,0.0230517139229017,0.00188559968790798,0.0143893389556470];

%

% FangChaOri = var(OriValue);

%

% FangChaRes = var(ResidualError);

%

% HouYanCha = FangChaRes / FangChaOri;

% %%%%%%%%%%%%%%%%%%%%%%%%%%%%%%The formula of posterior error%%%%%%%%%%%%%%%%%%%%%%%%%%%%%%%%

% OriValue = [17.91 , 17.89 , 17.39 , 17.65 , 17.61 , 17.35 , 16.75 , 17.86 , 17.46 , 17.66];

%

% ResidualError = [0,0.00582419039983506,-0.0820433435843846,0.148366858701863,-0.196795140998269,0.221487024903325,-0.219464304804692,0.190970950598430,-0.139443681318941,0.162606586029778];

%

% RelativeError = [0,0.000325555640013139,0.00471784609455921,0.00840605431738599,0.0111751925609466,0.0127658227609986,0.0131023465555040,0.0106926624075269,0.00798646513854186,0.00920762095298855];

%

% FangChaOri = var(OriValue);

%

% FangChaRes = var(ResidualError);

%

% HouYanCha = FangChaRes / FangChaOri;

% %%%%%%%%%%%%%%%%%%%%%%%%%%%%%%The formula of posterior error%%%%%%%%%%%%%%%%%%%%%%%%%%%%%%%%

% OriValue = [193510 , 190390 , 173730 , 147280 , 210610 , 175030 , 171540 , 175540 , 148140 , 171200 , 177530 , 181170];

%

% ResidualError = [0,-0.0919957496225834,-0.0393530579749495,-0.00680975642171688,-0.00162865599850193,0.0120916987361852,0.00237600962282158,-0.00425985862966627,-0.0120406854839530,-0.0175157518824562,-0.0188722294988111,-0.0290111690410413];

%

% RelativeError = [0,4.83196331858729e-07,2.26518494070969e-07,4.62368035151880e-08,7.73304210864597e-09,6.90835784504668e-08,1.38510529487092e-08,2.42671677661289e-08,8.12790973670378e-08,1.02311634827431e-07,1.06304452761849e-07,1.60132301380147e-07];

%

% FangChaOri = var(OriValue);

%

% FangChaRes = var(ResidualError);

%

% HouYanCha = FangChaRes / FangChaOri;

ResidualError = [0,-0.0919957496225834,-0.0393530579749495,-0.00680975642171688,-0.00162865599850193,0.0120916987361852,0.00237600962282158,-0.00425985862966627,-0.0120406854839530,-0.0175157518824562,-0.0188722294988111,-0.0290111690410413];

n = length(ResidualError);

ResidualErrorpf = ResidualError.^2;

MSE = sum(ResidualErrorpf) / n;
