# Supplementary material for: Calculation and realization of new method grey residual error correction model
Source: PLoS One. 2021 Jul 19;16(7):e0254154. doi: 10.1371/journal.pone.0254154 (PMC8289091; doi:10.1371/journal.pone.0254154)
Supplement: Attachment — Submitted filename: letter - Response to Reviewer2.doc [file pone.0254154.s004.doc]

Answer to Reviewer2 letter

May 10,2021

Dear reviewer,

I am very grateful to you for taking the time to read my article,and putting forward a lot of valuable suggestions for amendments. According to your suggestions, I will answer one by one as follows:

**Question1：**There is still room to improve English writing. Please improve English and engage native proof-reader if available.
There are too many grammatical errors, such as too many sentences without subject.“According to the characteristics of the original data, improve the gray prediction algorithm, optimize the background values, optimize the initial conditions, etc.”And please try more passive sentences.

**Answer1：**Thank the reviewer for their valuable suggestions. The revised manuscript has been revised for grammatical problems. The revised manuscript of this article has emphasized the stochastic volatility characteristics of the data, and the methods of optimizing the background value and optimizing the initial conditions are not effective in fitting the stochastic volatility characteristics. This article is modified to use the wavelet method to fit the residuals and the residual prediction function is established, and the residuals prediction function is used to correct the error caused by the GM method, and the WGM model is established. The WGM method achieves a very good fitting effect.

**Question2：** It is beneficial to cite other related publications in Plus One to reflect the relevance of your submission.

**Answer2：**Thank the reviewer for their valuable suggestions. This article has added relevant documents of PlosOne.

**Question3：**The amount of data is too small, it is limited for the examples of experimental analysis.

**Answer3：**Thank the reviewer for their valuable suggestions. The biggest advantage of the GM method is to predict unknown information based on a small amount of information. This is different from the big data method.

**Question4：** It will be helpful to add a flow-process diagram for describing the new residual correction grey model.

**Answer4：**Thank the reviewer for their valuable suggestions. The revised manuscript has added a flowchart of the improving GM method.

**Question5：** By the example data analysis, it can be found that the fitting accuracy and prediction effect of this method are better than the traditional GM(1,1) model. But how does it compare with other improved models?

**Answer5：**Thank the reviewer for their valuable suggestions. The revised paper mentions the FGM method in the literature [29]. The new WGM method studied in this article is compared with the FGM method and the traditional GM method. The experiment shows that the fitting accuracy and fitting effect of the WGM method are both Better than FGM method and GM method.

Kind regards,

 Lifang Xiao
