# Supplementary material for: Calculation and realization of new method grey residual error correction model
Source: PLoS One. 2021 Jul 19;16(7):e0254154. doi: 10.1371/journal.pone.0254154 (PMC8289091; doi:10.1371/journal.pone.0254154)
Supplement: Attachment — Submitted filename: Reviewer Comment.docx [file pone.0254154.s003.docx]

*The paper proposed a method to improve the prediction accuracy of the grey model based on the residual error correction method, and this new method (i.e., the improved GM method) was applied in three experimental analyses from the perspective of simulating the stock price of PetroChina, the population of US and the per capita energy consumption of China, respectively. Moreover, evaluation index including average residual, mean relative error and posterior difference ratio were selected, when compared the traditional GM method with the new method established by the residual correction simulation method. The effort of the authors is greatly appreciated, but in my opinion, Partial content needs to be improved, my comments are as follows:*

*1. It is obvious that the improved GM method is capable of addressing the issue of few samples and poor information in economy, management and engineering technology compared to the GM method. However, high requirements on the input data were not emphatic in this paper, considering the input data such as the stock price, the population and the per capita energy consumption present similar linear features and have the same sign. However, some input data present waveform features, or even highly non-linear features in reality, which was neglected in this paper. Therefore, input data selected in three experimental analyses should be representative and different, in other words, it is better to have three different characteristics of data rather than the same characteristics of data. As a consequence, emphasize the characteristics of the input data and change the type of the experimental analysis are necessary.*

*2. Similar to other prediction methods, the GM method also bears the limitations. Hence, the issue of improving the accuracy has received considerable critical attention. Nevertheless, literature and review involving introducing the advances in the GM method is lacking, especially the research progress of foreign scholars in this field. In addition, residual GM method mentioned is a common method to amend the traditional GM method and have been adopted in many aspects. Therefore, the innovation of this new improved GM method is insufficient. As a contrast, some other improved GM methods need to be introduced to* *highlight the advantages of the improved GM method in this paper.*

*3. It is worth mentioning that evaluation index in this paper have no explicit formula expressions. Except for average residual, mean relative error (MRE) and posterior difference ratio mentioned in this paper, other indicators such as mean absolute error (MAE), mean square error (MSE) and mean absolute percentage error (MAPE) should be taken into consideration. Moreover, the flow chart of constructing the improved GM method is necessary, given the complexity of the formula derivation.*

*Given the above three comments, I advise the paper need major changes, adding some important references bibliography at the same time.*
